# [8-(4-Phen­oxy­benzo­yl)-2,7-bis­(propan-2-yl­oxy)naphthalen-1-yl](4-phen­oxy­phen­yl)methanone

**DOI:** 10.1107/S1600536813000913

**Published:** 2013-01-16

**Authors:** Sayaka Yoshiwaka, Daichi Hijikata, Kosuke Sasagawa, Akiko Okamoto, Noriyuki Yonezawa

**Affiliations:** a Department of Organic and Polymer Materials Chemistry, Tokyo University of Agriculture & Technology, Koganei, Tokyo 184-8588, Japan

## Abstract

The entire title mol­ecule, C_42_H_36_O_6_, is completed by the application of a twofold axis. The 4-phen­oxy­benzoyl groups at the 1- and 8-positions of the naphthalene ring system are aligned almost anti­parallel. The dihedral angle between the best planes of the benzene rings of the benzoyl moieties and the naphthalene ring system is 70.52 (5)° and that between the best planes of the benzene rings of the phen­oxy groups and the naphthalene ring system is 27.80 (6)°. In the crystal, mol­ecules are linked into a three-dimensional architecture by C—H⋯O and C—H⋯π inter­actions.

## Related literature

For electrophilic aromatic aroylation of the naphthalene core, see; Okamoto & Yonezawa (2009 ▶); Okamoto *et al.* (2011 ▶). For the structures of closely related compounds, see: Hijikata *et al.* (2010 ▶); Sasagawa *et al.* (2012 ▶); Muto *et al.* (2010 ▶); Nakaema *et al.* (2008 ▶).
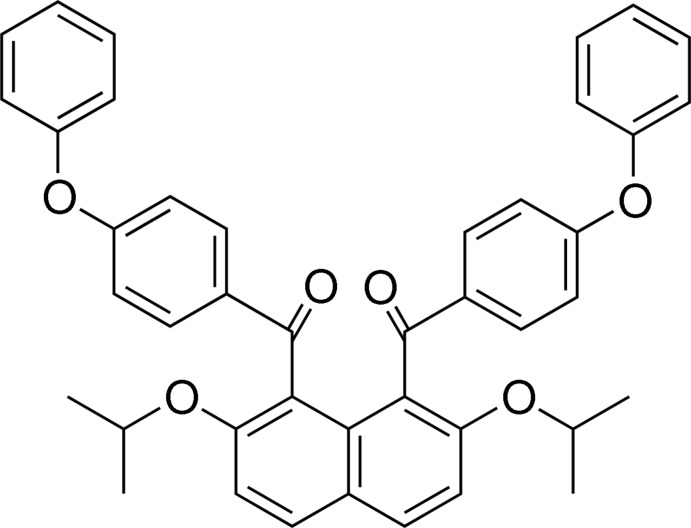


## Experimental

### 

#### Crystal data

C_42_H_36_O_6_
*M* *_r_* = 636.71
Monoclinic, 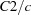

*a* = 22.7084 (4) Å
*b* = 10.3582 (2) Å
*c* = 14.7152 (3) Å
β = 100.106 (1)°
*V* = 3407.58 (11) Å^3^
*Z* = 4
Cu *K*α radiation
μ = 0.66 mm^−1^
*T* = 193 K
0.60 × 0.60 × 0.50 mm

#### Data collection

Rigaku R-AXIS RAPID diffractometer
Absorption correction: numerical (*NUMABS*; Higashi, 1999 ▶) *T*_min_ = 0.693, *T*_max_ = 0.734
28911 measured reflections
3101 independent reflections
2749 reflections with *I* > 2σ(*I*)
*R*_int_ = 0.029

#### Refinement

*R*[*F* ^2^ > 2σ(*F* ^2^)] = 0.036
*wR*(*F* ^2^) = 0.096
*S* = 1.04
3101 reflections
221 parameters
H-atom parameters constrained
Δρ_max_ = 0.21 e Å^−3^
Δρ_min_ = −0.16 e Å^−3^

### 

Data collection: *PROCESS-AUTO* (Rigaku, 1998 ▶); cell refinement: *PROCESS-AUTO*; data reduction: *PROCESS-AUTO*; program(s) used to solve structure: *Il Milione* (Burla *et al.*, 2007 ▶); program(s) used to refine structure: *SHELXL97* (Sheldrick, 2008 ▶); molecular graphics: *ORTEPIII* (Burnett & Johnson, 1996 ▶); software used to prepare material for publication: *SHELXL97*.

## Supplementary Material

Crystal structure: contains datablock(s) I, global. DOI: 10.1107/S1600536813000913/pk2459sup1.cif

Structure factors: contains datablock(s) I. DOI: 10.1107/S1600536813000913/pk2459Isup2.hkl

Supplementary material file. DOI: 10.1107/S1600536813000913/pk2459Isup3.cml

Additional supplementary materials:  crystallographic information; 3D view; checkCIF report

## Figures and Tables

**Table 1:** Hydrogen-bond geometry (Å, °) *Cg* is the centroid of the C8–C13 ring.

*D*—H⋯*A*	*D*—H	H⋯*A*	*D*⋯*A*	*D*—H⋯*A*
C12—H12⋯O1^i^	0.95	2.44	3.3398 (15)	158
C16—H16⋯*Cg* ^ii^	0.95	2.97	3.8383 (19)	152

Symmetry codes: (i) 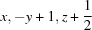; (ii) 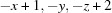.

**Fig. 1.**
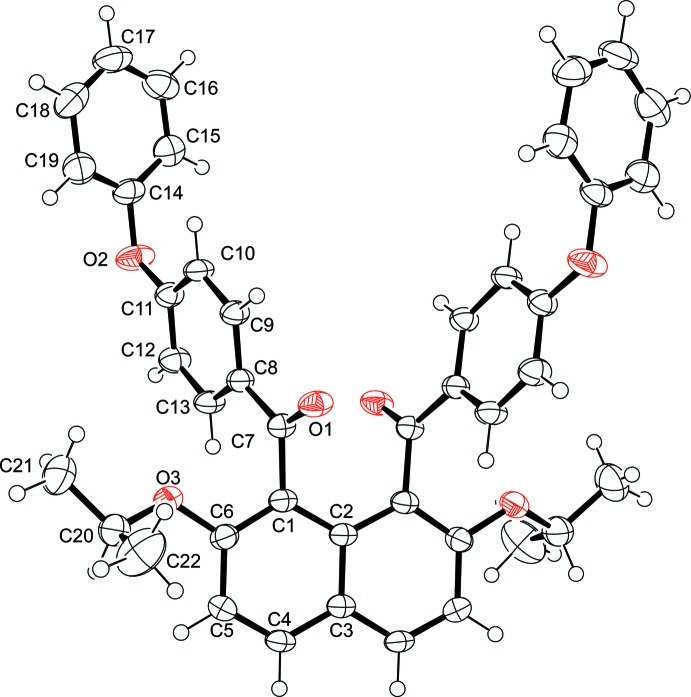
The molecular structure of compound (I). Displacement ellipsoids are drawn at the 50% probability level.

**Fig. 2.**
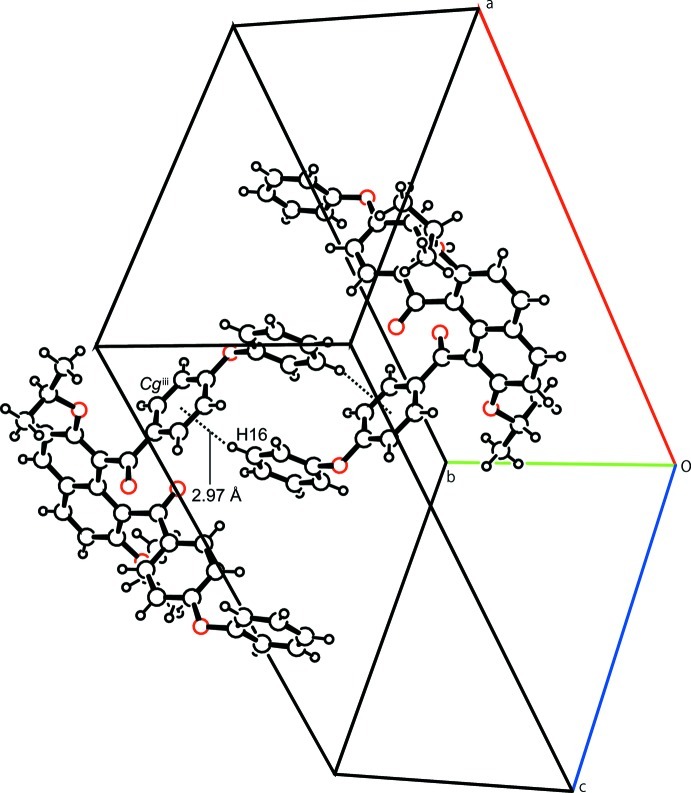
A partial packing diagram of compound (I), showing the intermolecular C—H···π interactions (dashed lines). Cg is the centroid of the C8—C13 ring. Symmetry code: (iii) 1 - x, -y, - z +2.

**Fig. 3.**
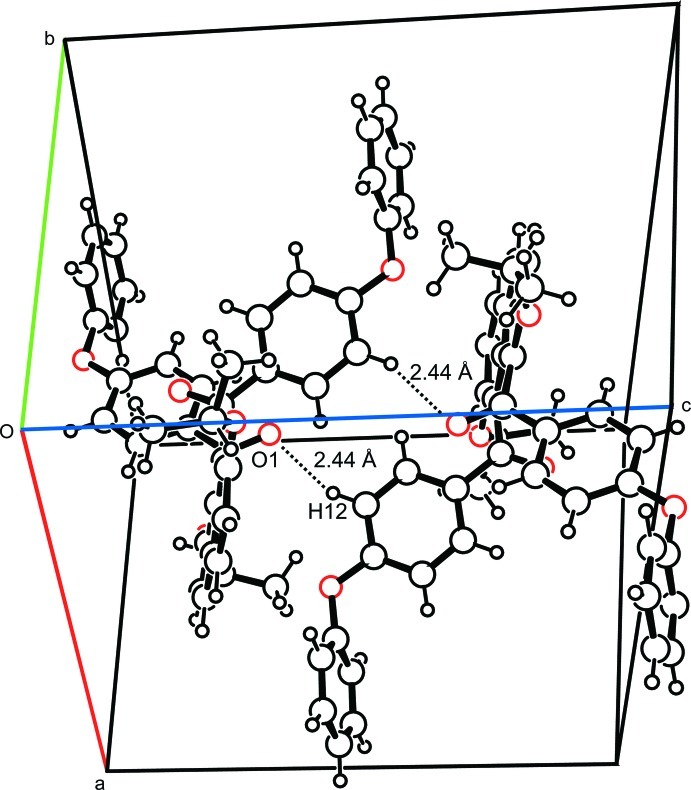
A partial packing diagram of compound (I), showing the C12–H12···O1 hydrogen interactions (dashed lines). Symmetry code: x, 1 - y, 1/2 + z.

## supplementary crystallographic information

### Comment

In the course of our study on electrophilic aromatic aroylation of the naphthalene ring core, 1,8-diaroylnaphthalene compounds have proved to be formed regioselectively by the choice of suitable acidic mediators (Okamoto & Yonezawa, 2009 Okamoto *et al.*, 2011). Recently, we have reported the crystal structures of several 1,8-diaroylated naphthalene analogues exemplified by 1,8-dibenzoyl-2,7-dimethoxynaphtalene (Nakaema *et al.*, 2008), [2,7-dimethoxy-8-(4-propylbenzoyl)naphthalene-1-yl]-(4-propylphenyl)methanone (Sasagawa *et al.*, 2012) and [2,7-dimethoxy-8-(4-methylbenzoyl)-1-naphthyl](4-methylphenyl)methanone (Muto *et al.* 2010). In the crystals of these compounds, two aroyl groups tend to attach to the naphthalene ring in nearly perpendicular manners and oriented in the opposite direction (*anti-*orientation). Recently, the crystal structure of 2,7-dimethoxy-1,8-bis(4-phenoxybenzoyl)naphthalene has been clarified to take *syn*-orientation, where two phenoxybenzoyl groups are positioned on the same side against the naphthalene ring plane (Hijikata *et al.* 2010). As a part of our continuing studies on the molecular structures of these kinds of homologous molecules, the X-ray crystal structure of the title compound *peri*-aroylnaphthalene bearing isopropoxy groups at the 2,7-positions is discussed in this article.

The molecular structure of the title compounds is displayed in Fig 1. Two 4-phenoxybenzoyl groups are situated in *anti*-orientation and are twisted away from the attached naphthalene ring. This molecule lies on a crystallographic 2-fold axis so that the asymmetric unit consists of one-half of the molecule. The dihedral angle between the best plane of the inner benzene ring of the 4-phenoxybenzoyl groups and the naphthalene system is 70.52 (5)°.

Centrosymmetrically related molecules are linked into dimeric unit by pairs of C—H···π interactions between the hydrogen atom (H16) on the terminal phenoxy group and the π-system of the benzene ring in the benzoyl moiety (C8–C13) (C16—H16···*Cg*^iii^, Fig. 2). The molecules of the title compound are aligned in an antiparallel fashion with the adjacent molecule. The terminal benzene ring of the phenoxybenzoyl group interacts with the inner benzene ring of the phenoxybenzoyl group of the adjacent molecule. Both of the pairs of the facing benzene rings in the couple of the phenoxybenzoyl groups are situated almost perpendicularly to the benzene ring in the benzoyl moiety (C8–C13). Then two identical interactions are formed to give cyclic structure between the two phenoxybenzoyl groups.

Furthermore, an oxygen atom of the carbonyl group forms intermolecular C—H···O interaction with the *m*-hydrogen of the benzoyl benzene ring of the other adjacent molecule (C12—H12···O1 = 2.44 Å, Fig. 3).

### Experimental

1,8-(4-phenoxybenzoyl)-2,7-dihydroxynaphtalene (0.3 mmol, 157 mg), tetrabutylammonium iodide (0.03 mmol, 113 mg), potassium carbonate (0.9 mmol, 127 mg) and DMF (0.75 ml) were placed into a 10 ml flask, followed by stirring at room temperature under nitrogen for 1 h. 2-Bromopropane (1.8 mmol, 224 mg) was to the solution and heated at 70 °C for 5 h. After cooling to room temperature, the reaction mixture was poured into ice-cold water (20 ml). The aqueous layer was extracted with ethyl acetate (20 ml × 2). The combined extracts were washed with 2 *M* aqueous NaOH followed by washing the brine. The extracts thus obtained were dried over anhydrous MgSO_4_. The solvent was removed under reduced pressure to give a cake (yield 22%). Colourless single crystals suitable for X-ray diffraction were obtained by repeated crystallization from methanol.

^1^H NMR *δ* (300 MHz, CDCl_3_): 1.04 (12*H*, d, *J* = 6.0 Hz), 4.51 (2*H*, sep, *J* = 6.0 Hz), 6.89 (4*H*, d, *J* = 8.1 Hz), 7.09 (4*H*, d, *J* = 8.1 Hz), 7.13 (2*H*, d, *J* = 9.0 Hz), 7.17 (2*H*, d, *J* = 8.1 Hz), 7.37 (4*H*, t, *J* = 8.1 Hz), 7.70 (4*H*, d, *J* = 8.1 Hz), 7.86 (2*H*, d, *J* = 9.0 Hz),

^13^C NMR *δ* (125 MHz, CDCl_3_): 21.6, 71.5, 113.1, 116.7, 120.0, 122.6, 124.1, 125.2, 129.8, 130.4, 131.3, 131.6, 134.3, 154.6, 155.8, 161.1, 196.0 p.p.m.

IR (KBr): 1656 (C=O), 1601, 1505, 1452 (Ar), 1267 (C—O—C) cm^-1^

HRMS (m/*z*): [*M*+H]^+^ called. for C_42_H_37_O_6_, 637.2581, found, 637.2590.

m.p. = 450.2–451.4 K

### Refinement

All H atoms were found in a difference map and were subsequently refined as riding atoms, with C–H = 0.95 (aromatic), 0.98 (methyl) and 1.00 (methine) Å and with *U*_iso_(H) = 1.2 *U*_eq_(C).

### Crystal data

**Table:**

C_42_H_36_O_6_	*F*(000) = 1344
*M**_r_* = 636.71	*D*_x_ = 1.241 Mg m^−^^3^
Monoclinic, *C*2/*c*	Cu *K*α radiation, λ = 1.54187 Å
Hall symbol: -C 2yc	Cell parameters from 26902 reflections
*a* = 22.7084 (4) Å	θ = 3.1–68.3°
*b* = 10.3582 (2) Å	µ = 0.66 mm^−^^1^
*c* = 14.7152 (3) Å	*T* = 193 K
β = 100.106 (1)°	Block, colorless
*V* = 3407.58 (11) Å^3^	0.60 × 0.60 × 0.50 mm
*Z* = 4	

### Data collection

**Table:**

Rigaku R-AXIS RAPID diffractometer	3101 independent reflections
Radiation source: rotaing anode	2749 reflections with *I* > 2σ(*I*)
Graphite monochromator	*R*_int_ = 0.029
Detector resolution: 10.000 pixels mm^-1^	θ_max_ = 68.3°, θ_min_ = 4.0°
ω scans	*h* = −27→27
Absorption correction: numerical (*NUMABS*; Higashi, 1999)	*k* = −12→12
*T*_min_ = 0.693, *T*_max_ = 0.734	*l* = −17→17
28911 measured reflections	

### Refinement

**Table:**

Refinement on *F*^2^	Secondary atom site location: difference Fourier map
Least-squares matrix: full	Hydrogen site location: inferred from neighbouring sites
*R*[*F*^2^ > 2σ(*F*^2^)] = 0.036	H-atom parameters constrained
*wR*(*F*^2^) = 0.096	*w* = 1/[σ^2^(*F*_o_^2^) + (0.0477*P*)^2^ + 1.7648*P*] where *P* = (*F*_o_^2^ + 2*F*_c_^2^)/3
*S* = 1.04	(Δ/σ)_max_ = 0.001
3101 reflections	Δρ_max_ = 0.21 e Å^−^^3^
221 parameters	Δρ_min_ = −0.16 e Å^−^^3^
0 restraints	Extinction correction: *SHELXL97* (Sheldrick, 2008), Fc^*^=kFc[1+0.001xFc^2^λ^3^/sin(2θ)]^-1/4^
Primary atom site location: structure-invariant direct methods	Extinction coefficient: 0.00263 (12)

### Special details

**Table:**

Geometry. All e.s.d.'s (except the e.s.d. in the dihedral angle between two l.s. planes) are estimated using the full covariance matrix. The cell e.s.d.'s are taken into account individually in the estimation of e.s.d.'s in distances, angles and torsion angles; correlations between e.s.d.'s in cell parameters are only used when they are defined by crystal symmetry. An approximate (isotropic) treatment of cell e.s.d.'s is used for estimating e.s.d.'s involving l.s. planes.
Refinement. Refinement of *F*^2^ against ALL reflections. The weighted *R*-factor *wR* and goodness of fit *S* are based on *F*^2^, conventional *R*-factors *R* are based on *F*, with *F* set to zero for negative *F*^2^. The threshold expression of *F*^2^ > σ(*F*^2^) is used only for calculating *R*-factors(gt) *etc*. and is not relevant to the choice of reflections for refinement. *R*-factors based on *F*^2^ are statistically about twice as large as those based on *F*, and *R*- factors based on ALL data will be even larger.

### Fractional atomic coordinates and isotropic or equivalent isotropic displacement parameters (Å^2^)

**Table:**

	*x*	*y*	*z*	*U*_iso_*/*U*_eq_	
O1	0.43879 (4)	0.44016 (8)	0.67336 (6)	0.0378 (2)	
O2	0.34771 (4)	0.63044 (8)	0.78791 (7)	0.0418 (3)	
O3	0.40230 (5)	0.16418 (9)	1.04457 (6)	0.0475 (3)	
C3	0.5000	0.83092 (15)	0.7500	0.0279 (4)	
C8	0.42720 (5)	0.40189 (11)	0.82730 (8)	0.0288 (3)	
C7	0.43753 (5)	0.48524 (11)	0.74958 (8)	0.0290 (3)	
C2	0.5000	0.69308 (15)	0.7500	0.0263 (3)	
C5	0.39876 (6)	0.83609 (12)	0.78052 (9)	0.0341 (3)	
H5	0.3651	0.8834	0.7926	0.041*	
C1	0.44642 (5)	0.62900 (11)	0.76338 (8)	0.0281 (3)	
C6	0.39740 (5)	0.70012 (11)	0.77734 (8)	0.0317 (3)	
C9	0.41352 (6)	0.27221 (11)	0.81026 (8)	0.0312 (3)	
H9	0.4104	0.2394	0.7493	0.037*	
C13	0.43221 (6)	0.44792 (11)	0.91753 (8)	0.0340 (3)	
H13	0.4417	0.5362	0.9301	0.041*	
C10	0.40443 (6)	0.19034 (11)	0.88074 (8)	0.0326 (3)	
H10	0.3947	0.1022	0.8683	0.039*	
C11	0.40974 (6)	0.23839 (12)	0.96986 (8)	0.0330 (3)	
C12	0.42366 (6)	0.36733 (12)	0.98865 (9)	0.0369 (3)	
H12	0.4272	0.3996	1.0498	0.044*	
C14	0.38932 (6)	0.03327 (12)	1.03096 (8)	0.0374 (3)	
C15	0.43487 (7)	−0.05536 (14)	1.05092 (9)	0.0429 (3)	
H15	0.4750	−0.0273	1.0699	0.052*	
C20	0.28915 (6)	0.68545 (14)	0.75933 (11)	0.0458 (4)	
H20	0.2858	0.7664	0.7951	0.055*	
C16	0.42179 (8)	−0.18549 (14)	1.04313 (10)	0.0509 (4)	
H16	0.4530	−0.2472	1.0576	0.061*	
C19	0.33118 (7)	−0.00546 (14)	1.00293 (11)	0.0475 (4)	
H19	0.2999	0.0563	0.9898	0.057*	
C17	0.36410 (8)	−0.22609 (14)	1.01469 (11)	0.0554 (4)	
H17	0.3553	−0.3157	1.0092	0.066*	
C18	0.31893 (8)	−0.13676 (16)	0.99415 (12)	0.0568 (4)	
H18	0.2790	−0.1650	0.9738	0.068*	
C21	0.24694 (7)	0.58531 (18)	0.78572 (15)	0.0677 (5)	
H21A	0.2491	0.5069	0.7492	0.102*	
H21B	0.2060	0.6190	0.7736	0.102*	
H21C	0.2583	0.5650	0.8515	0.102*	
C22	0.27768 (9)	0.7160 (2)	0.65854 (14)	0.0779 (6)	
H22B	0.3046	0.7852	0.6462	0.117*	
H22C	0.2361	0.7439	0.6398	0.117*	
H22A	0.2848	0.6388	0.6235	0.117*	
C4	0.44890 (5)	0.89855 (11)	0.76600 (8)	0.0310 (3)	
H4	0.4496	0.9903	0.7667	0.037*	

### Atomic displacement parameters (Å^2^)

**Table:**

	*U*^11^	*U*^22^	*U*^33^	*U*^12^	*U*^13^	*U*^23^
O1	0.0591 (6)	0.0264 (4)	0.0288 (5)	−0.0060 (4)	0.0102 (4)	−0.0024 (3)
O2	0.0344 (5)	0.0291 (5)	0.0639 (6)	−0.0017 (4)	0.0141 (4)	0.0045 (4)
O3	0.0838 (7)	0.0299 (5)	0.0307 (5)	−0.0094 (5)	0.0151 (5)	0.0025 (4)
C3	0.0356 (9)	0.0225 (8)	0.0252 (8)	0.000	0.0040 (7)	0.000
C8	0.0332 (6)	0.0226 (6)	0.0307 (6)	−0.0009 (5)	0.0062 (5)	−0.0004 (5)
C7	0.0330 (6)	0.0238 (6)	0.0300 (6)	−0.0019 (5)	0.0047 (5)	−0.0010 (5)
C2	0.0356 (9)	0.0211 (8)	0.0217 (8)	0.000	0.0041 (6)	0.000
C5	0.0372 (7)	0.0263 (6)	0.0402 (7)	0.0041 (5)	0.0102 (5)	−0.0019 (5)
C1	0.0368 (6)	0.0215 (6)	0.0260 (6)	−0.0007 (5)	0.0053 (5)	0.0009 (4)
C6	0.0351 (6)	0.0264 (6)	0.0342 (6)	−0.0033 (5)	0.0079 (5)	0.0005 (5)
C9	0.0414 (7)	0.0249 (6)	0.0282 (6)	−0.0028 (5)	0.0082 (5)	−0.0030 (5)
C13	0.0459 (7)	0.0220 (6)	0.0339 (7)	−0.0032 (5)	0.0071 (5)	−0.0037 (5)
C10	0.0445 (7)	0.0203 (6)	0.0336 (7)	−0.0039 (5)	0.0091 (5)	−0.0015 (5)
C11	0.0424 (7)	0.0276 (6)	0.0299 (6)	−0.0008 (5)	0.0090 (5)	0.0036 (5)
C12	0.0528 (8)	0.0301 (6)	0.0280 (6)	−0.0022 (6)	0.0079 (5)	−0.0035 (5)
C14	0.0569 (8)	0.0298 (6)	0.0274 (6)	−0.0047 (6)	0.0120 (6)	0.0054 (5)
C15	0.0485 (8)	0.0450 (8)	0.0350 (7)	−0.0007 (6)	0.0067 (6)	0.0031 (6)
C20	0.0350 (7)	0.0392 (7)	0.0631 (9)	−0.0008 (6)	0.0086 (6)	0.0002 (7)
C16	0.0712 (10)	0.0380 (8)	0.0445 (8)	0.0107 (7)	0.0131 (7)	0.0081 (6)
C19	0.0496 (8)	0.0442 (8)	0.0497 (8)	0.0024 (6)	0.0114 (7)	0.0080 (6)
C17	0.0856 (12)	0.0317 (7)	0.0525 (9)	−0.0101 (8)	0.0222 (8)	0.0046 (6)
C18	0.0566 (9)	0.0569 (10)	0.0590 (10)	−0.0201 (8)	0.0159 (8)	0.0014 (8)
C21	0.0414 (8)	0.0627 (11)	0.1020 (15)	−0.0074 (8)	0.0205 (9)	0.0098 (10)
C22	0.0671 (12)	0.0893 (14)	0.0687 (12)	−0.0250 (10)	−0.0120 (9)	0.0140 (11)
C4	0.0408 (7)	0.0190 (5)	0.0332 (6)	0.0015 (5)	0.0064 (5)	−0.0017 (5)

### Geometric parameters (Å, º)

**Table:**

O1—C7	1.2198 (14)	C11—C12	1.3891 (17)
O2—C6	1.3711 (14)	C12—H12	0.9500
O2—C20	1.4401 (16)	C14—C19	1.372 (2)
O3—C11	1.3760 (14)	C14—C15	1.376 (2)
O3—C14	1.3947 (15)	C15—C16	1.381 (2)
C3—C4	1.4101 (14)	C15—H15	0.9500
C3—C4^i^	1.4102 (14)	C20—C22	1.494 (2)
C3—C2	1.428 (2)	C20—C21	1.509 (2)
C8—C9	1.3916 (16)	C20—H20	1.0000
C8—C13	1.3966 (17)	C16—C17	1.370 (2)
C8—C7	1.4841 (16)	C16—H16	0.9500
C7—C1	1.5116 (16)	C19—C18	1.390 (2)
C2—C1^i^	1.4295 (13)	C19—H19	0.9500
C2—C1	1.4295 (13)	C17—C18	1.375 (2)
C5—C4	1.3586 (17)	C17—H17	0.9500
C5—C6	1.4094 (17)	C18—H18	0.9500
C5—H5	0.9500	C21—H21A	0.9800
C1—C6	1.3800 (17)	C21—H21B	0.9800
C9—C10	1.3829 (17)	C21—H21C	0.9800
C9—H9	0.9500	C22—H22B	0.9800
C13—C12	1.3788 (18)	C22—H22C	0.9800
C13—H13	0.9500	C22—H22A	0.9800
C10—C11	1.3881 (17)	C4—H4	0.9500
C10—H10	0.9500		
			
C6—O2—C20	119.65 (10)	C19—C14—O3	119.69 (13)
C11—O3—C14	118.75 (10)	C15—C14—O3	119.09 (13)
C4—C3—C4^i^	120.42 (15)	C14—C15—C16	119.40 (14)
C4—C3—C2	119.79 (7)	C14—C15—H15	120.3
C4^i^—C3—C2	119.79 (7)	C16—C15—H15	120.3
C9—C8—C13	118.63 (11)	O2—C20—C22	111.41 (14)
C9—C8—C7	118.90 (10)	O2—C20—C21	104.39 (12)
C13—C8—C7	122.45 (10)	C22—C20—C21	113.15 (15)
O1—C7—C8	121.21 (10)	O2—C20—H20	109.3
O1—C7—C1	118.46 (10)	C22—C20—H20	109.3
C8—C7—C1	120.33 (10)	C21—C20—H20	109.3
C3—C2—C1^i^	117.67 (7)	C17—C16—C15	120.36 (15)
C3—C2—C1	117.67 (7)	C17—C16—H16	119.8
C1^i^—C2—C1	124.67 (14)	C15—C16—H16	119.8
C4—C5—C6	119.00 (11)	C14—C19—C18	118.71 (14)
C4—C5—H5	120.5	C14—C19—H19	120.6
C6—C5—H5	120.5	C18—C19—H19	120.6
C6—C1—C2	120.06 (11)	C16—C17—C18	119.81 (14)
C6—C1—C7	116.97 (10)	C16—C17—H17	120.1
C2—C1—C7	122.40 (11)	C18—C17—H17	120.1
O2—C6—C1	115.93 (10)	C17—C18—C19	120.58 (15)
O2—C6—C5	122.41 (11)	C17—C18—H18	119.7
C1—C6—C5	121.65 (11)	C19—C18—H18	119.7
C10—C9—C8	120.99 (11)	C20—C21—H21A	109.5
C10—C9—H9	119.5	C20—C21—H21B	109.5
C8—C9—H9	119.5	H21A—C21—H21B	109.5
C12—C13—C8	121.08 (11)	C20—C21—H21C	109.5
C12—C13—H13	119.5	H21A—C21—H21C	109.5
C8—C13—H13	119.5	H21B—C21—H21C	109.5
C9—C10—C11	119.28 (11)	C20—C22—H22B	109.5
C9—C10—H10	120.4	C20—C22—H22C	109.5
C11—C10—H10	120.4	H22B—C22—H22C	109.5
O3—C11—C10	123.51 (11)	C20—C22—H22A	109.5
O3—C11—C12	115.70 (11)	H22B—C22—H22A	109.5
C10—C11—C12	120.79 (11)	H22C—C22—H22A	109.5
C13—C12—C11	119.24 (11)	C5—C4—C3	121.77 (11)
C13—C12—H12	120.4	C5—C4—H4	119.1
C11—C12—H12	120.4	C3—C4—H4	119.1
C19—C14—C15	121.12 (13)		
			
C9—C8—C7—O1	−5.73 (18)	C9—C8—C13—C12	−0.21 (19)
C13—C8—C7—O1	172.58 (12)	C7—C8—C13—C12	−178.51 (12)
C9—C8—C7—C1	173.94 (11)	C8—C9—C10—C11	−0.71 (19)
C13—C8—C7—C1	−7.76 (17)	C14—O3—C11—C10	0.68 (19)
C4—C3—C2—C1^i^	−177.89 (7)	C14—O3—C11—C12	−178.86 (12)
C4^i^—C3—C2—C1^i^	2.11 (7)	C9—C10—C11—O3	−179.14 (12)
C4—C3—C2—C1	2.11 (7)	C9—C10—C11—C12	0.38 (19)
C4^i^—C3—C2—C1	−177.89 (7)	C8—C13—C12—C11	−0.1 (2)
C3—C2—C1—C6	−1.15 (12)	O3—C11—C12—C13	179.58 (12)
C1^i^—C2—C1—C6	178.85 (12)	C10—C11—C12—C13	0.0 (2)
C3—C2—C1—C7	169.94 (8)	C11—O3—C14—C19	−84.84 (16)
C1^i^—C2—C1—C7	−10.06 (8)	C11—O3—C14—C15	98.74 (15)
O1—C7—C1—C6	110.54 (13)	C19—C14—C15—C16	−0.4 (2)
C8—C7—C1—C6	−69.13 (14)	O3—C14—C15—C16	175.98 (12)
O1—C7—C1—C2	−60.81 (15)	C6—O2—C20—C22	60.29 (17)
C8—C7—C1—C2	119.52 (11)	C6—O2—C20—C21	−177.26 (13)
C20—O2—C6—C1	−150.33 (12)	C14—C15—C16—C17	0.8 (2)
C20—O2—C6—C5	29.72 (18)	C15—C14—C19—C18	−0.5 (2)
C2—C1—C6—O2	178.90 (9)	O3—C14—C19—C18	−176.89 (13)
C7—C1—C6—O2	7.34 (16)	C15—C16—C17—C18	−0.3 (2)
C2—C1—C6—C5	−1.14 (17)	C16—C17—C18—C19	−0.7 (2)
C7—C1—C6—C5	−172.70 (11)	C14—C19—C18—C17	1.1 (2)
C4—C5—C6—O2	−177.55 (11)	C6—C5—C4—C3	−1.49 (18)
C4—C5—C6—C1	2.49 (19)	C4^i^—C3—C4—C5	179.19 (13)
C13—C8—C9—C10	0.62 (18)	C2—C3—C4—C5	−0.81 (13)
C7—C8—C9—C10	178.99 (11)		

Symmetry code: (i) −*x*+1, *y*, −*z*+3/2.

### Hydrogen-bond geometry (Å, º)

Cg is the centroid of the C8–C13 ring.

**Table:**

*D*—H···*A*	*D*—H	H···*A*	*D*···*A*	*D*—H···*A*
C12—H12···O1^ii^	0.95	2.44	3.3398 (15)	158
C16—H16···*Cg*^iii^	0.95	2.97	3.8383 (19)	152

Symmetry codes: (ii) *x*, −*y*+1, *z*+1/2; (iii) −*x*+1, −*y*, −*z*+2.

## References

[bb1] Burla, M. C., Caliandro, R., Camalli, M., Carrozzini, B., Cascarano, G. L., De Caro, L., Giacovazzo, C., Polidori, G., Siliqi, D. & Spagna, R. (2007). *J. Appl. Cryst.* **40**, 609–613.

[bb2] Burnett, M. N. & Johnson, C. K. (1996). *ORTEPIII* Report ORNL-6895. Oak Ridge National Laboratory. Tennessee, USA.

[bb3] Higashi, T. (1999). *NUMABS* Rigaku Corporation, Tokyo, Japan.

[bb4] Hijikata, D., Takada, T., Nagasawa, A., Okamoto, A. & Yonezawa, N. (2010). *Acta Cryst.* E**66**, o2902–o2903. 10.1107/S1600536810042170 PMC3009209 21589079

[bb5] Muto, T., Kato, Y., Nagasawa, A., Okamoto, A. & Yonezawa, N. (2010). *Acta Cryst.* E**66**, o2752. 10.1107/S1600536810039620 PMC3009178 21588956

[bb6] Nakaema, K., Watanabe, S., Okamoto, A., Noguchi, K. & Yonezawa, N. (2008). *Acta Cryst.* E**64**, o807. 10.1107/S1600536808007009 PMC2961268 21202298

[bb7] Okamoto, A., Mitsui, R., Oike, H. & Yonezawa, N. (2011). *Chem. Lett.* **40**, 1283–1284.

[bb8] Okamoto, A. & Yonezawa, N. (2009). *Chem. Lett.* **38**, 914–915.

[bb9] Rigaku (1998). *PROCESS-AUTO* Rigaku Corporation, Tokyo, Japan.

[bb11] Sasagawa, K., Hijikata, D., Sakamoto, R., Okamoto, A. & Yonezawa, N. (2012). *Acta Cryst.* E**68**, o2596. 10.1107/S1600536812033582 PMC3415032 22905019

[bb12] Sheldrick, G. M. (2008). *Acta Cryst.* A**64**, 112–122. 10.1107/S0108767307043930 18156677

